# Integrating advanced analytical methods to assess epigenetic marks affecting response to hypomethylating agents in higher risk myelodysplastic syndrome

**DOI:** 10.1186/s10020-025-01123-7

**Published:** 2025-02-14

**Authors:** Theodoros Nikolopoulos, Eleftherios Bochalis, Theodora Chatzilygeroudi, Vasiliki Chondrou, Irene Dereki, Katerina Athanasopoulou, John Zafeiropoulos, Kyriakos Bourikas, George P. Patrinos, Argiris Symeonidis, Argyro Sgourou

**Affiliations:** 1 Biology Laboratory, School of Science and Technology, Hellenic Open University, Patras, Greece; 2 Chemistry Laboratory, School of Science and Technology, Hellenic Open University, Patras, Greece; 3 School of Health Sciences, Faculty of Medicine, Hematology Division, University of Patras, Patras, Greece; 4 Division of Hematological Malignancies, Department of Oncology, Sidney Kimmel Comprehensive Cancer Center, Baltimore, MD USA; 5 Laboratory of Pharmacogenomics and Inaffiliationidualized Therapy, Department of Pharmacy, School of Health Sciences, University of Patras, University Campus, Rion, Patras Greece; 6 College of Medicine and Health Sciences, Department of Genetics and Genomics, United Arab Emirates University, Al Ain, Abu Dhabi, UAE; 7 Zayed Center for Health Sciences, United Arab Emirates University, Al Ain, Abu Dhabi, UAE; 8 Clinical Bioinformatics Unit, Department of Pathology, Faculty of Medicine and Health Sciences, Erasmus University Medical Center, Rotterdam, The Netherlands

**Keywords:** Myelodysplastic syndromes, Hypomethylating agents, Response assessment, DNA methylation, LC-MS/MS analysis, MeD-seq data analysis

## Abstract

**Background:**

Patients with higher-risk (HR) myelodysplastic syndrome (MDS), ineligible for allogeneic hematopoietic stem cell transplantation (alloHSCT), require prompt therapeutic interventions, such as treatment with hypomethylating agents (HMAs) to restore normal DNA methylation patterns, mainly of oncosuppressor genes, and consequently to delay disease progression and increase overall survival (OS). However, response assessment to HMA treatment relies on conventional methods with limited capacity to uncover a wide spectrum of underlying molecular events.

**Methods:**

We implemented liquid chromatography-tandem mass spectrometry (LC-MS/MS) to assess 5’ methyl-2’ deoxycytidine (5mdC), 5’ hydroxy-methyl-2’-deoxycytidine (5hmdC) levels and global adenosine/thymidine ([dA]/[T]) ratio in bone marrow aspirates from twenty-one HR MDS patients, pre- and post-HMA treatment. Additionally, targeted methylation analysis was performed by interpretation of NGS-methylation (MeD-seq) data obtained from the same patient cohort.

**Results:**

LC/MS-MS analysis revealed a significant hypomethylation status in responders (Rs), already established at baseline and a trend for further DNA methylation reduction post-HMA treatment. Non-responders (NRs) reached statistical significance for DNA hypomethylation only post-HMA treatment. The 5hmdC epigenetic mark was approximately detected at 37.5–40% among NRs and Rs, implying the impairment of the natural active demethylation pathway, mediated by the ten-eleven (TET) 5mdC dioxygenases. R and NR subgroups displayed a [dA]/[T] ratio < 1 (0.727 − 0.633), supporting high frequences of 5mdC transition to thymidine. Response to treatment, according to whole genome MeD-seq data analysis, was associated with specific, scattered hypomethylated DMRs, rather than presenting a global effect across genome. MeD-seq analysis identified divergent epigenetic effects along chromosomes 7, 9, 12, 16, 18, 21, 22, X and Y. Within statistically significant selected chromosomal bins, genes encoding for proteins and non-coding RNAs with reversed methylation profiles between Rs and NRs, were highlighted.

**Conclusions:**

Implementation of powerful analytical tools to identify the dynamic DNA methylation changes in HR MDS patients undergoing HMA therapy demonstrated that LC-MS/MS exerts high efficiency as a broad-based but rapid and cost-effective methodology (compared to MeD-seq) to decode different perspectives of the epigenetic background of HR MDS patients and possess discriminative efficacy of the response phenotype to HMA treatment.

## Introduction

Myelodysplastic syndromes (MDS) are clonal hematopoietic stem cell disorders, mainly characterized by abnormal development and maturation of hematopoietic progenitor cells in the bone marrow, resulting in peripheral blood cytopenias and by an increased tendency of disease progression towards acute myelogenous leukemia (AML). Morphological examination of the bone marrow typically reveals dysplastic changes in the hematopoietic cell precursors indicating abnormal cell proliferation and maturation, leading to an heterogeneous clonal development (Li et al. 2022). At the molecular level MDS patients exhibit not only DNA-based genetic abnormalities in their hematopoietic progenitors, but also aberrations related to the cell membrane immunophenotype, gene expression, epigenetic profile and bone marrow microenvironment (Maggioni and Della Porta 2023; Alaggio et al. 2022; Kouroukli et al. 2022). Comprehensive patient profiling necessitates the use of extended next-generation sequencing (NGS) panels to evaluate mutation status, providing critical insights into prognosis and therapy selection. Additionally, multiparameter flow cytometry can contribute to diagnosis and is increasingly established in the monitoring of disease response, further enhancing precision in disease management (Abdulbaki and Pullarkat 2024; Tobiasson and Kittang 2019; Verigou et al. 2024). Currently, with the increasing application of interventional treatment approaches, assessment of minimal residual disease (MRD) is the recommended method for response assessment in AML patients and its potential role in the evaluation and monitoring of HR MDS patients is being explored (Shih et al. 2023).

The study of MDS has revealed the significant role of genetic and epigenetic alterations in the pathophysiology of this disease. Mutations affecting genes responsible for epigenetic modifications, such as *DNMT3A*,* TET2*, and *ASXL1* not only are prevalent among the elderly population but they are also considered as founding and driver mutations in MDS(Stengel et al. 2021; Risques and Kennedy 2018; Papaemmanuil et al. 2013). These mutations are early molecular events in the pathophysiology of the disease, since they are also present in patients with clonal hematopoiesis of indeterminate potential (CHIP). Clonal expansions evolve over time, particularly in individuals, aged above 55 years. Thus, clones with *DNMT3A* and *TP53* mutations have slower growth (∼ 5% per year), whereas mutations such as *SRSF2P95H* grow over 50% per year and *TET2*-mutant clones emerge across all ages(Fabre et al. 2022). Apart from mutation involvement in bone marrow clonogenicity, aberrant DNA methylation patterns are also very common in the course of MDS however, their contribution to disease development and progression has not yet been fully elucidated. In cancer biology, the widespread genome hypomethylation is associated with genomic instability and activation of transposable elements and increased oncogene expression (Besselink et al. 2023; Grundy et al. 2022), both of which can contribute to MDS development and progression. On the contrary, specific genes, involved in hematopoietic differentiation, apoptosis, and cell cycle regulation, such as Cyclin Dependent Kinase Inhibitor 2B (*CDKN2B* or *P15*)(Au et al. 2003; Raj et al. 2007), adenosine 5’-Monophosphoramidase (*HINT1*)(Iwai et al. 2005) and gene sets including *P15*(Shen et al. 2010), have been detected in hypermethylated mode and silenced, often associated with adverse clinical outcomes in MDS patients.

The integration of epigenome information into existing prognostic models may improve risk stratification and guide treatment decisions for MDS patients, since certain methylation signatures have been associated with disease progression, transformation to AML and decreased OS(Khan et al. 2013; Cabezon et al. 2021). Higher-risk (HR) MDS group requires prompt treatment decisions, consideration of interventional therapies, such as hypomethylating agents (HMAs) and, in fit patients, allogeneic hematopoietic stem cell transplantation (alloHSCT). HMAs and particularly the cytosine analogs azacytidine (5-azacytidine) and decitabine (5-aza-2′-deoxycytidine) are common treatment options for HR MDS patients, aiming to slow down abnormal clonal growth and restore peripheral blood cell counts(Hellstrom-Lindberg and Kroger 2023). However, only 30–50% of HR MDS patients respond to HMAs, after a median of 4–5 months post-treatment start initiation(Fenaux et al. 2009; Laribi et al. 2019) and a minority of MDS patients experience prolonged (> 24 months) responses (Zeidan et al. 2018). Although the high diversity in the response to HMA treatment has been extensively reported, the specific factors contributing to the induction of a favorable response still remain to a substantial degree, undetermined. Moreover, the epigenetic changes in clonal marrow cells, as well as the correlation of these changes with patient response or secondary resistance to HMAs and AML progression, have not been fully elucidated (Pleyer and Greil 2015). Although some authors have observed a significant association between HMA-induced hypomethylation and both, hematological response and overall survival(Shen et al. 2010; Yang et al. 2006), uncertainty remains regarding the prognostic value of hypomethylation induction(Khan et al. 2013; Voso et al. 2014). Additionally, although abnormal DNA methylation has been primarily studied in the context of CpG islands within gene promoters in MDS and AML (Chatzilygeroudi et al. 2024), CpG islands constitute only a small fraction of the methylome. Genome-wide methylation studies, using bone marrow samples from patients before and after HMAs treatment with HMAs are limited in the literature.

**Fig. 1:**
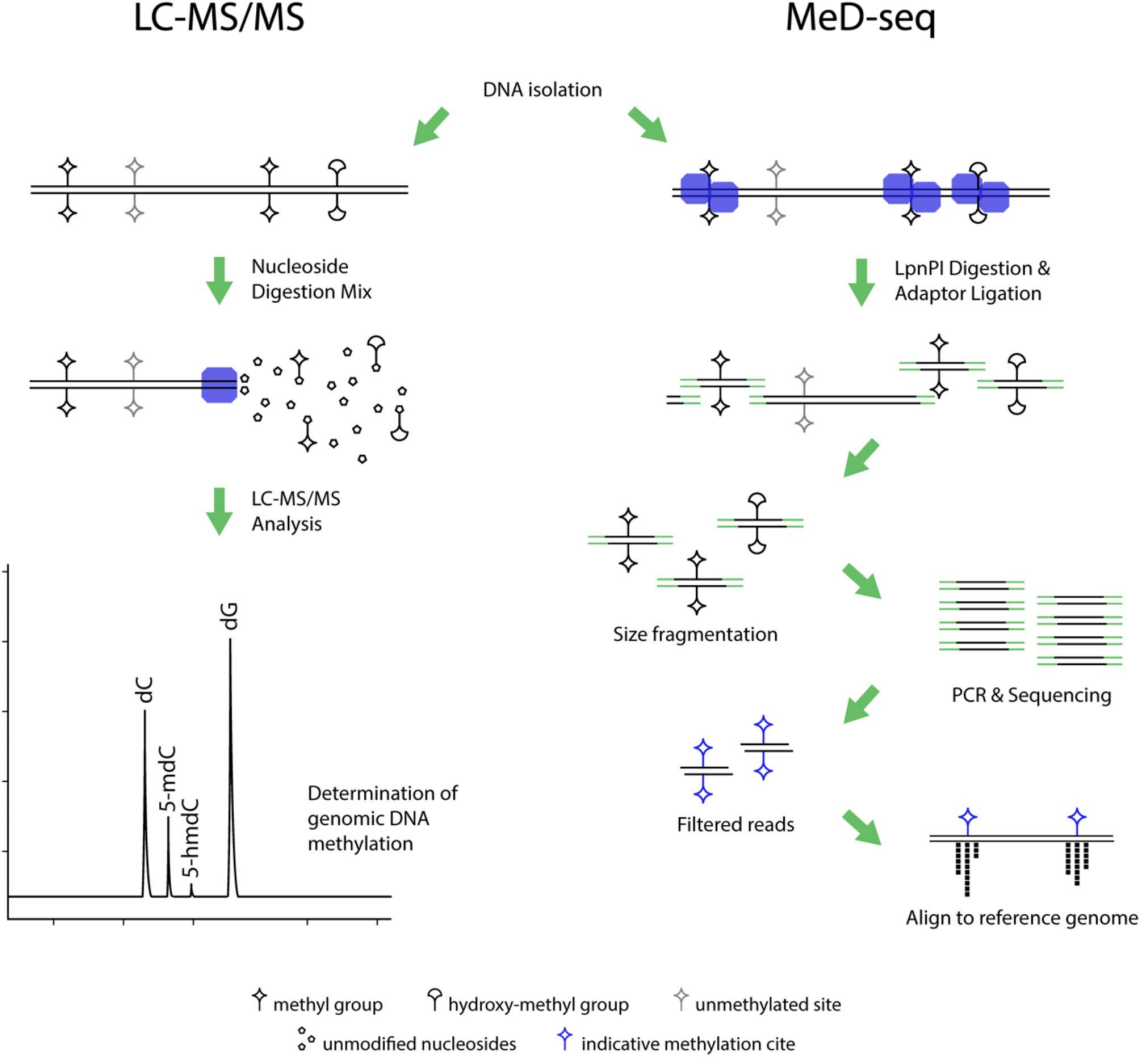
LC-MS/MS analysis (left) vs MeD-seq methodology (right). In **LC-MS/MS** analysis DNA is hydrolyzed into nucleosides, which are then separated by liquid chromatography and quantified by mass spectrometry. By this method different forms of deoxy cytosine modifications, such as 5mdC and 5hmdC can be detectable and distinguished in genomic DNA in contrast to MeD-seq or other conventional NGS analysis that prerequisites DNA digestion with methylation-sensitive enzymes or bisulfite conversion. LC-MS/MS is considered a method of high sensitivity and specificity, however provides information only about universal cytosine modifications rather than site-specific DNA methylation patterns. **MeD-seq** method is based on next-generation sequencing (NGS) technology and provides genome-wide profiling of DNA methylation patterns. DNA is first treated with methylation-dependent restriction enzyme LpnPI and then is subjected to NGS. Computational analysis is finally performed to determine methylation status at specific genomic loci. By this method genome-wide, base-resolution DNA methylation profiling is provided. Limitations are the first-step enzyme digest which can introduce bias, especially in regions with high GC content and secondly the bioinformatics expertise requirements for data analysis. Another limitation is the indispensable higher costs compared to LC-MS/MS. 5mdC: 5’ methyl- 2’-deoxycytidine, 5hmdC: 5’ hydroxy-methyl- 2’-deoxycytidine

In the present study, we have conducted a comprehensive evaluation of genomic DNA methylation status in twenty-one HR MDS patients, prior and post-HMA-treatment and the same analysis was performed in seven healthy controls. Global 5’ methyl-2’ deoxycytidine (5mdC) and 5’ hydroxy-methyl 2’-deoxycytidine (5hmdC) epigenetic marks, as well as chromosomal methylation distribution and targeted methylation signatures (DMRs) were assessed to enable discrimination between responders from non-responders to HMA therapy. Further to methylation analysis we have compared the randomness of spontaneous deamination of 5mdC to thymidine in HR MDS patients versus controls to estimate potential increase in the mutagenesis rate, attributed to HMA-treatment. Two complementary highly sensitive approaches, liquid chromatography-tandem mass spectrometry (LC-MS/MS) and NGS methylation assay (MeD-seq) (Fig. 1), have been implemented to address the above queries, taking into consideration their relevant advantages and limitations.

## Materials and methods

### MDS patient and healthy control cohorts

In total, 21 patients with HR MDS, exerting ≤ 20% bone marrow blasts, received treatment with azacytidine (AZA) or decitabine (DAC) by standard scheduling (75mg/m^2^ × 7 days, 28-day cycle for AZA and 20mg/m^2^ × 5 days, 28-day cycle, for DAC) at the University Hospital of Patras. All patients provided their written informed consent according to the Declaration of Helsinki, being informed about both clinical and translational investigations and the study was approved by the University General Hospital of Patras Ethics & Scientific Committee (approval decision number 33807/24.12.2020).

Treatment responders (R) versus non-responders (NR) were classified according to International Working Group (IWG) response criteria (marrow complete remission, mCR) for HR MDS patients, released in 2006(Cheson et al. 2006). mCR category acknowledges meaningful improvements in bone marrow characteristics (e.g., blast reduction) without demanding normalization of peripheral blood counts. This response criterion is particularly useful in patients who may not achieve full hematologic recovery but show significant disease modification at the marrow level. Bone marrow aspirates were collected from the whole patient’s cohort before treatment initiation, as well as at the evaluation of response (after 5–7 treatment cycles, 6–8 months post-ΗΜΑ treatment initiation). Peripheral blood from 7 elderly donors with matching age and gender was collected and represented the control group of samples for the LC-MS/MS analytical method. Peripheral blood samples provide an adequate picture of the bone marrow environment since, high concordance of variant detection and gene expression profile between peripheral blood and bone marrow has been repeatedly reported (Osman et al. 2023; Zeidan et al. 2023; Tsekoura et al. 2023). Mononuclear cells (lymphocytes and monocytes) from both, peripheral blood (PBMCs) and bone marrow aspirates (BMMCs) were isolated by Ficoll gradient centrifugation, washed twice with 1X Phosphate Buffered Saline (PBS) and the purified cell pellet was used for genomic DNA extraction. Genomic DNA with simultaneous enzymatic removal of RNA by RNAse/DNAse (1.5 mU/μL RNase, Merck, KGaA, Darmstadt, Germany) was extracted using phenol: chloroform: isoamyl alcohol in 25:24:1 ratio (Sigma-Aldrich Pty Ltd, Merck KGaA, Darmstadt, Germany) and subjected to downstream analysis. Characteristics of the patient and control cohorts participating in this study are summarized in Table 1.

**Table 1:** List of MDS patients and healthy control samples. MDS samples were collected pre- and post-HMA treatment. (C1-C7: control DNA samples, R1-R9: responders to HMA according to mCR response, NR1-NR12: non-responders to HMA treatment)

A/A	Healthy control (C)/ Responders (*R*)/Non Responders (NR)	Male (M)/Female (F)	Age	AZA/DAC treated	Tissue BM/PB
1	C1	M	70	-	PB
2	C2	M	70	-	PB
3	C3	M	75	-	PB
4	C4	M	85	-	PB
5	C5	M	70	-	PB
6	C6	M	70	-	PB
7	C7	M	75	-	PB
8	R1	M	60	AZA	BM
9	R2	M	64	AZA	BM
10	R3	M	57	AZA	BM
11	R4	M	69	AZA	BM
12	R5	M	76	AZA	BM
13	R6	M	76	AZA	BM
14	R7	M	68	AZA	BM
15	R8	F	78	AZA	BM
16	R9	M	79	AZA	BM
17	NR1	M	79	DAC	BM
18	NR2	M	75	AZA	BM
19	NR3	M	82	AZA	BM
20	NR4	M	85	AZA	BM
21	NR5	M	69	AZA	BM
22	NR6	M	70	AZA	BM
23	NR7	M	61	AZA	BM
24	NR8	M	73	DAC	BM
25	NR9	M	84	DAC	BM
26	NR10	M	69	AZA	BM
27	NR11	M	83	AZA	BM
28	NR12	M	66	AZA	BM

### LC-MS/MS analysis of genomic DNA

Liquid chromatography-mass spectrometry technique combines Liquid Chromatography (LC) with a triple quadrupole (QqQ) mass spectrometer (LCMS-8050 system, Shimadzu, Japan). DNA samples from HR MDS were analyzed pre- and post- HMA treatment and compared with control samples for baseline methylation pre-treatment. Prior to LC-MS/MS analysis 400 ng of genomic DNA was digested and dephosphorylated by a Nucleoside Digestion Mix (NEB#M0649) to generate single nucleosides for further quantitative analysis, following manufacturer’s instructions. Reactions were purified with a special centrifugal filter 3 kDa MWCO Amicon^®^ Ultra (Merck Millipore).

LC-MS/MS system features a heated Electrospray Ionization (ESI) system and Multiple Reaction Monitoring (MRM) capabilities with high sensitivity and high speed. The column oven was set at 35 °C. A Shim-pack Scepter C18-120 column (4.6 mm x 100 mm, 5 μm, Shimadzu) with a 4.6 mm pre-column was used for the separation of nucleosides. The mobile phase was passed through the column by gradient elution with acidified H_2_O (1% CH_3_COOH) (solvent A) and acetonitrile (ACN) (solvent B). The flow rate was set at 0.2 ml/min with the solvent ratio starting at 95% A / 5% B and reaching 35% A / 65% B at 15 min. The total analysis time was 20 min and the injection volume was 10 μL (62.5ng DNA per injection). Samples were analyzed as duplicates or triplicates. Mass spectrometry detection was performed under positive electrospray ionization (ESI) mode. Nucleosides and derivatives were monitored by multiple reaction monitoring (MRM) modes using the mass transitions (precursor ions → product ions) of dG (deoxy guanosine) (268.4 → 152.4), 5-mdC (242.1 → 126.1), 5-hmdC (258.1 → 142.1), T (thymidine) (243.3 → 127.2) and dA (deoxyadenosine) (252.4 → 136.2)(Song et al. 2005). Calibration curves of 5-mdC and 5-hmdC were constructed by plotting the peak area ratios of 5-mdC/dG and 5-hmdC/dG versus the molar ratios of 5-mdC/10^2^dG and 5-hmdC/10^5^dG, respectively, based on data obtained from LC-ESI-MS/MS analysis. Calibration curves were also constructed for T and dA (Tang et al. 2015). Linearity was within the concentration range 0.4–6 for 5-mdC/10^2^dG and 60–500 for 5-hmdC/10^5^dG, with a coefficient of determination (R^2^) greater than 0.99. Results for 5mdC, 5hmdC, T and dA are given as mean values derived from four independent runs.

Statistical analysis was performed using SPSS software version 20. To assess statistical differences of 5mdC levels among control and patients’ groups before HMA treatment, a one-way ANOVA test with Bonferroni multiple comparison post hoc test was used. Paired t-test was performed to identify 5mdC differences before and after treatment between each MDS patient group. Normal distribution of data was assessed by the Shapiro-Wilk test. *P*-values less than 0.05 were considered statistically significant.

### MeD-seq data analysis

Methylated DNA sequencing (MeD-seq) is a high-throughput methodology that constitutes the targeted capture of differentially methylated regions (DMRs) across genome (whole genome approach). A methylation sensitive restriction enzyme digests into recognition sites of methylated over unmethylated cytosines, and DNA fragments are then subjected to NGS (Boers et al. 2018) (Fig. 1).

Raw sequencing files for 13 MDS samples pre- and post-HMA treatment (26 samples, R2, R3, R6-R9, NR1, NR2, NR8-NR12, Table 1) were retrieved from the SRA database under: PRJNA1075483 (https://www.ncbi.nlm.nih.gov/bioproject/1075483). The raw data were processed as described in (Boers et al. 2018), with the key steps summarized below.

The Universal Illumina adaptor was trimmed from the data and reads were filtered based on the presence of the LpnPI restriction site. Filtered reads were aligned to the hg38 human genome and were annotated for Genes (Transcription Start Sites, Gene bodies, and Transcription End Sites) and CpG islands using annotations from ENSEMBL. Differentially Methylated Region (DMR) detection was performed between two data sets (pre- and post-HMA treatment) for each MDS sample containing the regions of interest. Required filtering criteria for DMRs were the following: q-value less than 0.05, fold change greater than 2, and inclusion of at least 20 LpnPI recognition sites to ensure selection of genomic areas enriched in CGs. DMRs present in alternative and random genomic contigs were excluded from this analysis. DMRs were retrieved by using the χ^2^ test on read counts. Significance was called by either Bonferroni or FDR using the Benjamini-Hochberg procedure. Using this pipeline a methylation ratio is obtained for each DMR indicating an increase or decrease in methylation post-HMA patient’s treatment.

To reduce the right skewness of the data and mitigate the presence of outliers, log_10_ -transformation was performed on the methylation ratios for each region, since they possess only positive values that span a wide range. Post log_10_-transformations, positive and negative methylation values indicated increase and decrease in methylation status within the studied area, respectively.

To synthesize a comprehensive methylation profile for each chromosome, DMRs were split into two groups: those with increased methylation (upwards) and those with decreased methylation (downwards). A weighted mean methylation ratio was computed separately for each response category using log_10_-transformed methylation ratios, thus accommodating the bimodal distribution characteristic of the methylation data.

The composite weighted mean for each chromosome (WM_chr_) was derived using the equation:

A $$ W{M_{chr}}{\rm{ }} = {\rm{ }}\left( {{m_{u}} \times {\rm{ }}{w_{u}}} \right){\rm{ }} + {\rm{ }}\left( {{m_d} \times {\rm{ }}{w_{d}}} \right) $$

where *WM*_*chr*_​ represents the total weighted mean methylation for a given chromosome, *m*_*u*_​ is the mean methylation level of the upwards methylated regions, *w*_*u*​_ is the proportion of upwards methylated DMRs to the total number of DMRs within the chromosome, *m*_*d*_​ is the mean methylation level of the downwards methylated regions, and *w*_*d*_​ is the proportion of downwards methylated DMRs relative to the chromosome’s total DMR count. This calculation provided a weighted average methylation value that reflects the overall methylation status while accounting for the distribution and abundance of methylated regions across the chromosome. Also, for each category a weighted standard error was calculated for each chromosome (WSE_chr_) using the following equation:

B $$ \:WS{E_{chr}} = \sqrt {(SEM_u^2\: \times \:{w_u})\: + \:(SEM_d^2\: \times \:{w_d})} $$

where *WSE*_*chr*_ represents the total weighted standard error for a given chromosome, $\:{\text{SEM}}_{\text{u}}$ and $\:{\text{SEM}}_{\text{d}}$ represent the standard errors of the upwards and downwards methylated regions, respectively. $\:{\text{w}}_{\text{u}}$ and $\:{\text{w}}_{\text{d}}$ represent the weights as defined in equation [A].

To enable a more targeted and comprehensive analysis, the hg38 human genome was segmented into bins of 100,000 base pairs, with each DMR assigned to a bin based on coverage. To assess the genomic methylation profiles of Rs and NRs, the Savitzky-Golay (Savitzky and Golay 1964) filter was applied to smooth methylation trends for each category, enhancing the visualization of underlying patterns. To identify statistically significant differences in methylation levels across genomic bins and chromosomes between the R and NR groups of HR MDS patients, the Mann-Whitney U test—a robust non-parametric method well-suited for the bimodal distribution of methylation data—was utilized. Additionally, the magnitude of these differences was quantified using Cliff’s delta (δ) only for the statistically significant chromosomes. Interpretation of δ values was as follows:|δ| < 0.147 indicated negligible difference between categories, 0.147 ≤|δ| < 0.33 represented a small difference, 0.33 ≤|δ| < 0.474 denoted a medium change, and|δ| ≥ 0.474 signified a large change. This comprehensive approach provided both statistical and practical insights into the genomic methylation differences between R and NR groups.

## Results

### Detection of diverse epigenetic marks (5mdC/5hmdC) by LC-MS/MS

LC-MS/MS is considered as a gold standard method for the global analysis of DNA methylation derivatives (5mdC and 5hmdC) in human cancers compared to enzymatic digests or bisulfite treatment of DNA prior to NGS reactions, which fail to discriminate between 5mdC and 5hmdC, both of which are detected as 5mdC (Fig. 1)(Chowdhury et al. 2017).

**Fig. 2:**
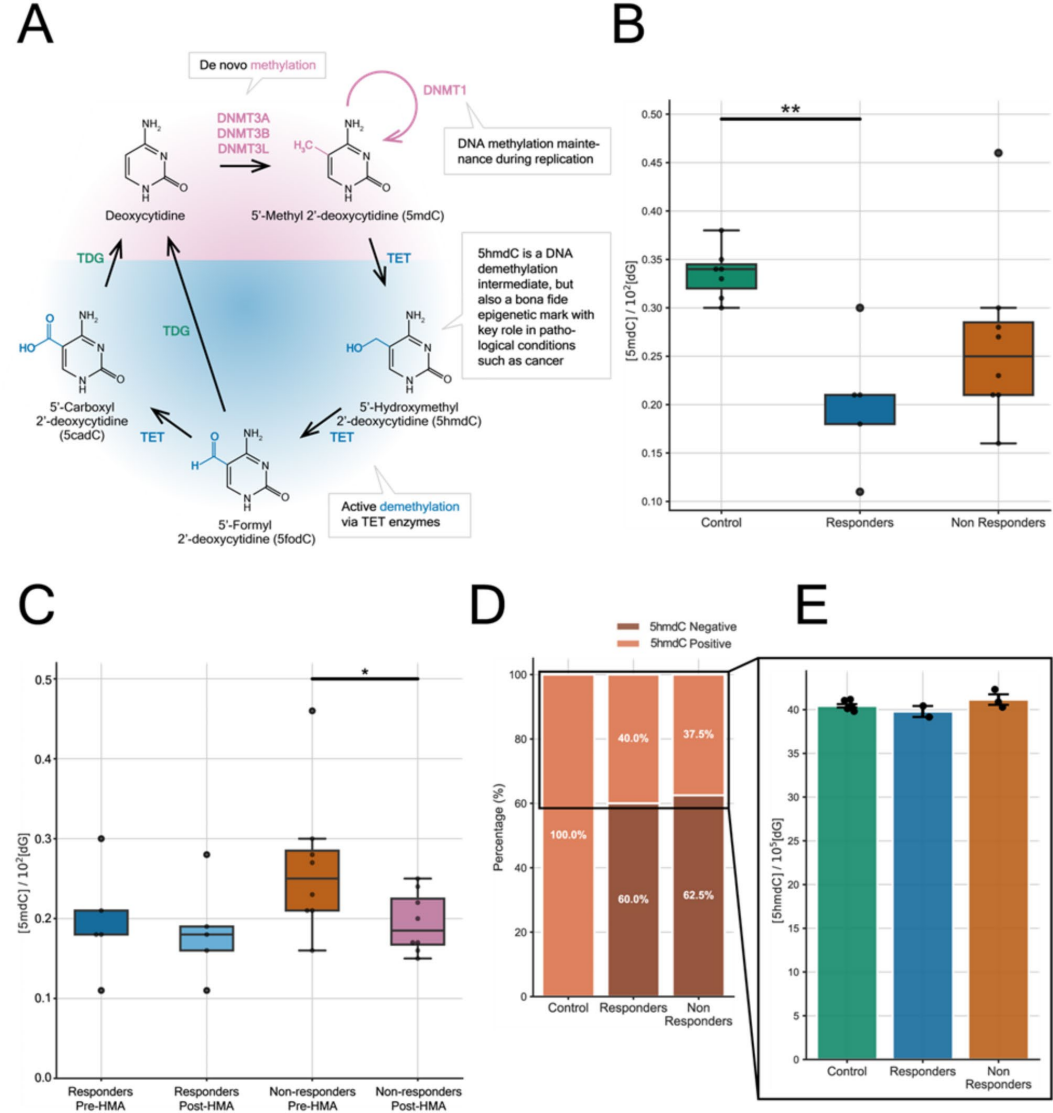
**A**) Maintenance of deoxy cytosine (followed by deoxy guanosine, CpG) methylation during DNA replication is provided by DNA methyl transferase DNMT1 whereas, DNMT3A/3B catalyzes the same reaction de novo at accessible genomic sites. Active demethylation pathway is mediated by the TET enzymes. Passive 5hmdC loss in cancer cells is reported to be the consequence of the high replication rate of such cells. **B**) Abundance of 5mdC among different HR MDS groups (R and NR) and healthy controls (C) pre-HMA treatment. Lower 5mdC levels among Rs and NRs compared to controls imply a hypomethylation status at baseline. Rs are hypomethylated at baseline with statistically significant values (** *p* < 0.01). **C**) Distribution of 5mdC levels pre- and post-HMA treatment within R and NR group of HR MDS cohort. Rs maintained at comparable levels of methylation post-HMA treatment in contrast to NRs, who significantly reduced methylation levels globally (* *p* < 0.05). Data are presented as boxplots including maximum and minimum values, the median value of each data set, outliers (°) and significant *p*-values. **D**) Percentage of HR MDS patients’ and healthy control samples presenting positive 5hmdC mark. 5hmdC appeared consistently in all healthy samples (100%), but approximately in 37.5–40% of HR MDS samples. **E**). 3Rs and 4NRs exhibited the 5hmdC mark both pre- and post-HMA treatment. C, R and NR indicate healthy controls, responders and non-responders respectively. Error bars depict the standard error present in the calculation of the global 5hmdC levels for each MDS category. HR MDS: High-risk MDS, 5mdC: 5’ methyl-2’ deoxycytidine, 5hmdC: 5’ hydroxy-methyl- 2’ deoxycytidine

In the present study, levels of 5’ methyl-2’ deoxycytidine (5mdC) and 5’ hydroxy-methyl 2’ deoxycytidine (5hmdC) residues, prior to and post-HMA treatment, were estimated by LC/MS-MS and further compared between MDS patients and the independent group of healthy controls (Table 1; Fig. 2). Total 5mdC across genomic DNA was calculated by the equation: [5mdC]/10^2^[dG] (further details in materials and methods section). Concentration of deoxy guanosine [dG] was selected as internal standard against [dC], based on the assumption that [dG] = {[dC] + [5mdC] + [5hmdC] + [other C modifications]} in genomic DNA. Therefore, [dG] is considered a unique and more accurate value rather than measurement of the independent cytosine modified nucleosides as a sum: {[dC] + [5mdC] + [5hmdC] + [other C modifications]}, potentially leading to experimental errors. Many of these cytosine derivatives are below detection limits of the method and additionally, guanosine modifications are much less prevalent in genomic DNA compared to methylated cytosines and its derivatives(Song et al. 2005).

To correlate global DNA methylation profiles (5mdC levels) among the different samples and in relation to HMA treatment response, MDS patients were categorized after clinical monitoring as responders (R1-9, Table 1) or non-responders (NR1-12, Table 1), according to mCR response. Although a trend for global hypomethylated status was documented for both R and NR groups compared to healthy controls (C1-7, Table 1), statistical significance was demonstrated only between controls and Rs (*p* = 0.014) (Fig. 2B). Comparison between the NR and R groups of HR MDS patients post-HMA treatment (Fig. 2C) revealed an actual significance for 5mdC lower values only within the NR group (*p* = 0.029). The R group yielded comparable 5mdC values pre- and post-HMA treatment.

A similar calibration curve, as previously described, was also applied for the 5hmdC calculation, but with a 10^5^ as a divisor ([5hmdC]/10^5^[dG]), since 5hmdC is represented at a frequency approximately ∼10–100-fold lower than 5mdC. Among HR MDS patients’ cohort, only 40% of Rs and 37,5% of NRs displayed detectable 5hmdC pre- and post-HMA treatment, even when analyzed in the high concentration mode (Fig. 2D). On the contrary, 5hmdC was consistently documented in all healthy control samples (Fig. 2E). This observation implies a potential dysregulation of α-ketoglutarate-dependent cytosine dioxygenases (TET1-3 enzymes), implicated in the natural biochemical DNA demethylation pathway by which 5mdC finally reverses to C, with 5hmdC representing the first product along the oxidative reaction pathway.

### Deviation of adenosine: thymidine ratio (≤ 1) highlights the frequent spontaneous deamination of 5mdC to thymidine

The ratio of adenosine (A) to thymidine (T) in double stranded DNA is expected to be approximately 1:1 due to the complementary base pairing in double helix DNA structure. Deviations in the human genome from 1:1 [dA]/[T] ratio often result from the spontaneous deamination reaction of the modified cytosine 5mdC that produces thymidine, which is unrecognizable and unable to be corrected by repairing enzymatic complexes that monitor the human genome for non-complementary bases. This reaction, if not corrected, converts a C-G base pair to a T-A during DNA replication. The deamination of 5mdC to thymidine is a significant source of mutations in DNA and leads to a deviating [dA]/[T] ratio.

**Fig. 3:**
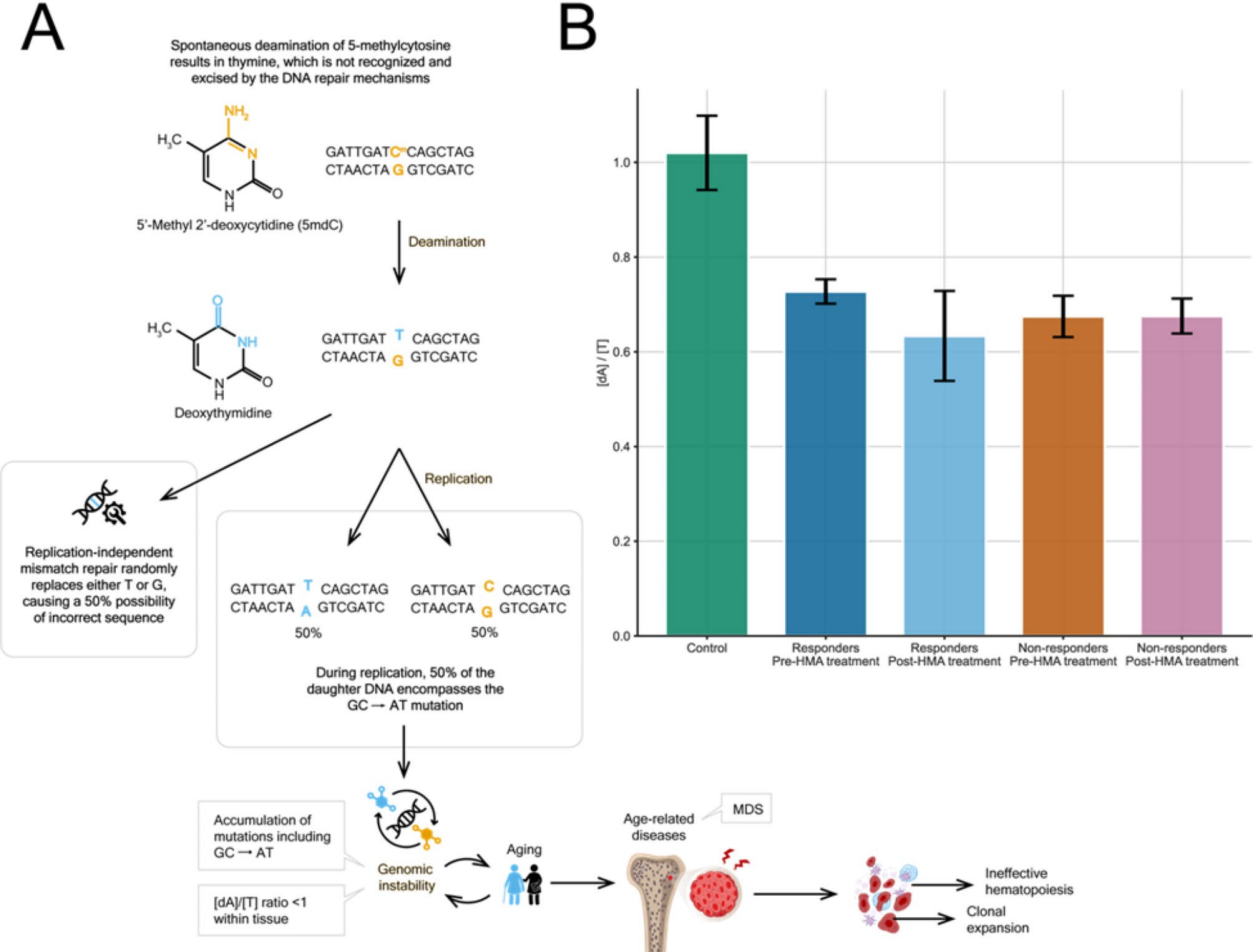
(**A**) Accumulation of GC→AT mutation, which is facilitated by the spontaneous deamination of 5mdC to thymine (T) is often reported in malignancies. Mutational overload accompanied with other epigenetically driven alterations are frequent in the elderly population and are prone to the development of age-related disorders, such as MDS. (**B**) [dA]/[T] ratio among Rs and NRs at baseline and post-HMA treatment, compared to healthy controls. Rs and NRs deviated from [dA]/[T] = 1, in contrast to healthy controls. Values at baseline and post-HMA treatment ranged between 0.727 − 0.633 with insignificant differences between R and NR subgroups of HR MDS, indicating a high mutational rate at baseline, not associated with HMA therapy. Error bars depict the standard error present in the calculation of the mean [dA]/[T] ratio for each category. R: responders, NR: non-responders, HR MDS: High-risk MDS

To this end we have comprehensively estimated the global [dA]/[T] ratio across HR MDS, depicted among Rs and NRs, pre- and post-HMA treatment, and healthy control samples (Fig. 3B). Calibration curves were plotted independently for A and T with values retrieved from LC-MS/MS analysis from escalating concentrations of Adenosine and Thymidine standards, respectively. The mean value of [dA]/[T] in healthy samples was 1.02 as expected. Rs and NRs pre- and post-HMA treatment constantly displayed [dA]/[T] ratios < 1 (0.727 − 0.633). Excluding the possibility of having RNA contamination that could skew the [dA]/[T] ratio, the establishment of newly introduced 5mdC to T transitions during DNA replication suggests that the recorded nucleotide imbalance reflects the presence of clonal genetic variation in the HR MDS bone marrow.

These results highlight the pre-existing deviation from normal values of thymine [T] concentration in HR MDS patients, already at baseline exempting effects derived from HMA treatment. The observed [dA]/[T] ratios < 1 in HR MDS samples implies increased levels of [T], attributed to deamination of 5mdC to thymidine and therefore, leading to the reduction of [dA]/[T] ratio. The potential of increased deamination rates of 5mdC to thymidine was clearly demonstrated by LC-MS/MS analysis and may account for the generation of mutations within CpG islands, flanking genetic loci and exerting transcriptional regulatory properties.

### Chromosome-wide mapping of DNA methylation patterns derived from MeD-seq analysis

MeD-seq facilitates the comprehensive analysis of genome-wide CpG methylation patterns. The methylation-sensitive restriction enzyme LpnPI, targets tetranucleotides containing methylated or hydroxymethylated CG dinucleotides, cleaving the DNA at 16 nucleotides downstream of the enzyme recognition site (Fig. 1). In the present study MeD-seq data from 13 h MDS patients (constituting a representative part of our MDS cohort) pre- and post-treatment with HMAs, were retrieved and further interpreted. Patients were sub-categorized into two independent groups: 6 responders (Rs) and 7 non-responders (NRs) to HMA therapy.

**Fig. 4:**
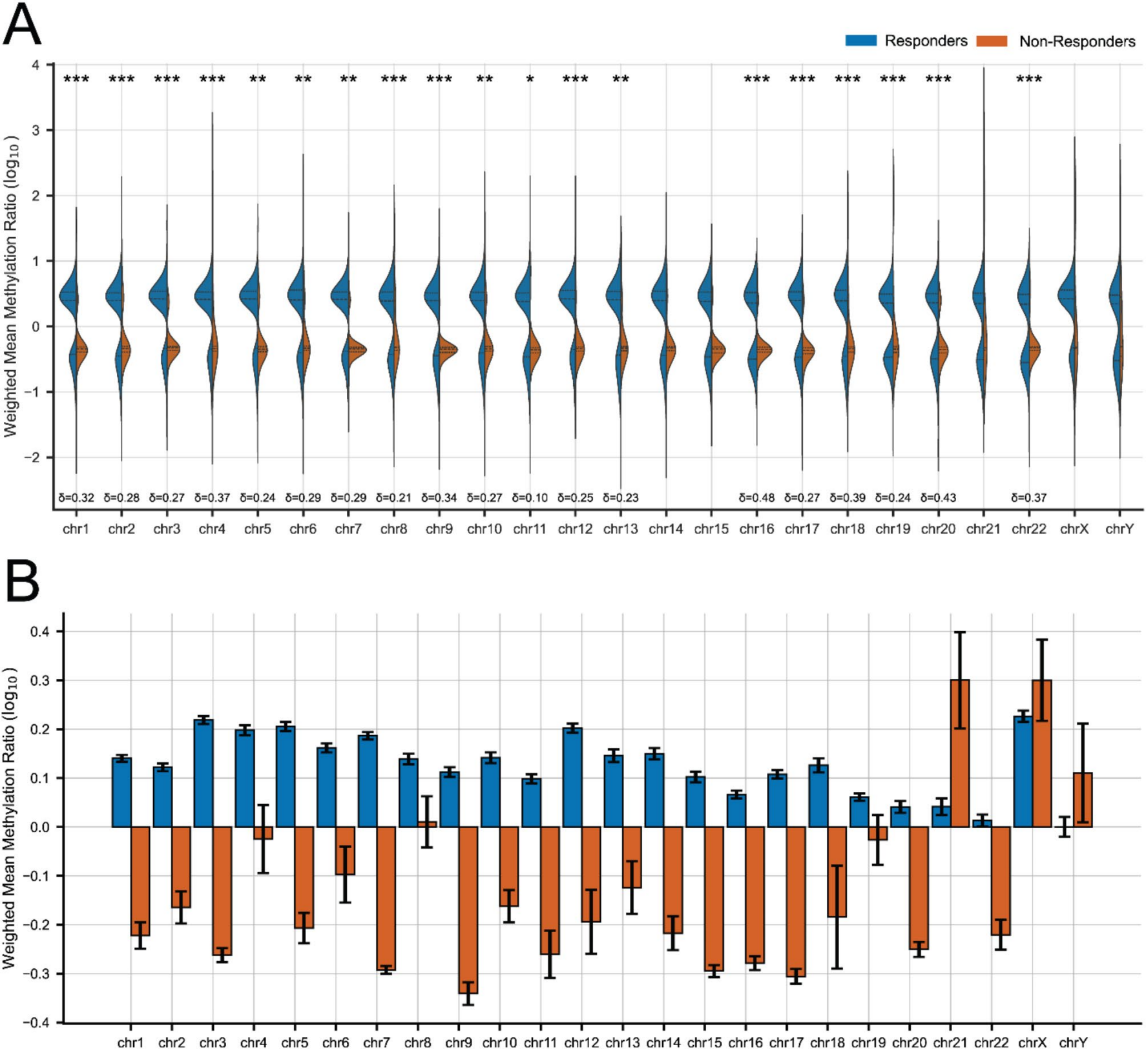
Α) Violin plots depict chromosomal methylation profiles by response category among HR MDS samples. Dotted lines inside the violin plots present the first quartile, median and third quartile values. Differentially methylated regions (DMRs) identified within each subgroup (R and NR) of HR MDS patients were catalogued by chromosome, and their methylation profiles were visualized using violin plots, which depict the distribution of methylation levels post-log_10_ transformation, with chromosomal location specified on the x-axis and methylation intensity on the y-axis. The shape of the plots reflects the density of methylation at various levels, revealing prevalent methylation states within R or NR subgroup. Asterisks represent the *p*-value calculated from the Mann-Whitney U-test, where * *p* < 0.05, ** *p* < 0.01 and *** *p* < 0.001. At the bottom of each violin plot Cliff’s delta (δ) is represented showing the magnitude of Rs hypermethylation compared to NRs. **B**) Bar plots showcasing the weighted average of methylation for each chromosome across subgrouped (R and NR) HR MDS patients according to HMA-response. Error bars depict the weighted standard error present in the calculation of the weighted average of methylation for each chromosome. Rs exhibit positive methylation values post-HMA treatment, in contrast to NRs who show mainly hypomethylation profiles in all autosomal chromosomes apart from chromosome 21 and both chromosomes X and Y. R: responders, NR: non-responders, HR MDS: High-risk MDS

For each human chromosome a weighted mean of methylation was calculated using the equation [A] (materials and methods section). The weighted mean provides a robust way for calculating total chromosomal methylation by utilizing the log_10_-transformed methylation ratios as well as by considering their respective weights. By assigning weights to each data point based on their significance or relevance, the weighted mean ensures that these differences are appropriately considered in the calculation. The equation [A] was applied to both R and NR MDS patients, who exhibited bimodal methylation distributions on each chromosome, documenting the presence of DMRs with both increased and decreased methylation associated with HMA treatment. Results are represented as violin plots displayed for each chromosome separately (Fig. 4A) to effectively summarize and visualize the distribution, central tendency, and spread of hypo- and hypermethylation. Rs demonstrated a more balanced bimodal distribution of methylation changes, slightly favoring regions with increased methylation, as indicated by the blue peak within the range of 0 to + 1. On the contrary, NRs showed an asymmetric pattern of chromosomal methylation distribution, where the majority followed a modest reduction in methylation, as evidenced by the red density peak between 0 and − 1. Chromosomes 7, 9, 12, 16, 18, and 22 exhibited statistically significant skewing (*p* < 0.05) towards regions with reduced methylation, with limited representation of regions showing increased methylation. Notably, chromosome 16 displayed a pronounced degree of hypermethylation in Rs compared to NRs, with a substantial effect size (Cliff’s delta = 0.48).

Moreover, the NR subgroup exhibited a substantial proportion of regions undergoing extreme hypermethylation, with methylation elevation ranging approximately from 100 to 1000-fold (corresponding to log_10_ values of 2 to 3). The significant methylation increase of these specific regions is also highlighted in Fig. 4B, underscoring a marked rise in methylation levels that surpass the distribution of hypomethylated regions on chromosomes 21, X, and Y. However, the limited number of differentially methylated regions (DMRs) on these chromosomes poses a challenge for statistical analysis, restricting the ability to draw definitive conclusions. Future studies should prioritize increasing the resolution of DMR detection to validate these findings and explore their biological significance.

In the bar chart provided (Fig. 4B), the distribution of weighted mean methylation values further corroborates these findings. The chart displays a notable differential methylation pattern between Rs and NRs, indicating potential epigenetic distinctions correlating with the HMA response categories. For the majority of chromosomes, Rs exhibited positive mean methylation values, indicating an increase in methylation post-HMA treatment, contrasting with the negative values observed in NRs, which are in line with results obtained from respective LC-MS/MS analysis, exerting a globally reduced methylation status. Conclusively, DNA methylation patterns across chromosomes is highly variable, underscoring the intricate nature of epigenetic regulation in relation to phenotypic outcomes.

### Targeted methylation analysis by MeD-seq reveals significant chromosomal regions discriminating responders from non-responders to HMA-therapy

Further analysis of MeD-seq data was performed to decode methylation profiles of targeted chromosomal regions. Genomic MeD-seq methylation data were divided into discrete segments or genomic regions of 100 kb in size, defined as chromosomal bins, that facilitate analysis and interpretation of genomic data by providing a systematic framework for organizing and comparing genomic features across different regions of the genome. The sequencing reads obtained from MeD-seq experiment were assigned to the appropriate bins based on their genomic coordinates.

**Fig. 5:**
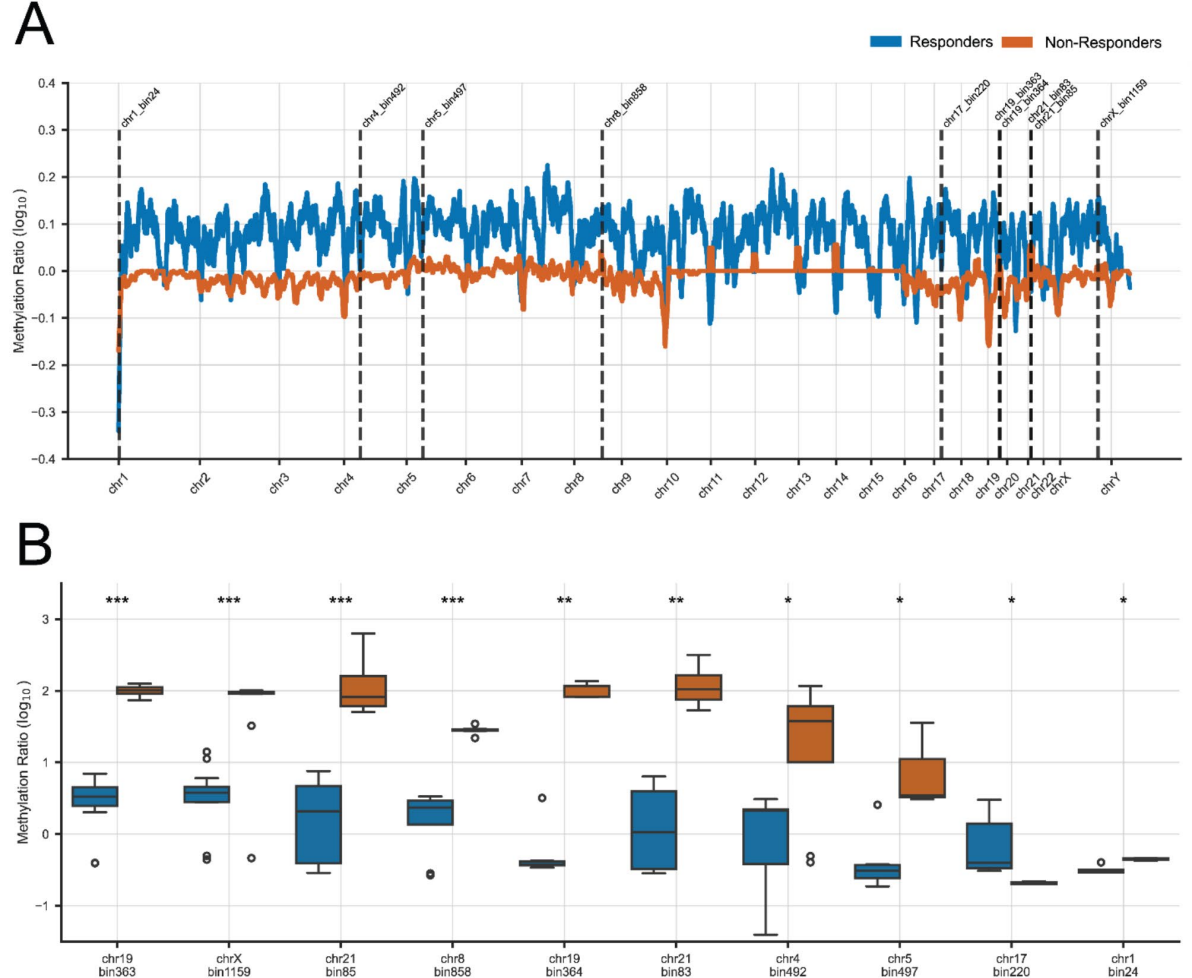
Α) Comparison of the genomic methylation profile between responders and non-responders. An average log_10_ methylation ratio is depicted in relation to the genomic bins. Specific statistically significant bins are highlighted with dotted lines. **Β)** Box plots depict log_10_-transformed methylation ratios across selected chromosomal bins with the highest statistical significance. Rs and NRs to HMA-treatment can be discriminated via their reversed methylation profiles within common chromosomal bins. Bins are indicated on the x-axis, and log_10_ methylation ratios on the y-axis. Statistical significance was determined by the Mann-Whitney U test, which is indicated by asterisks above each bin (*p ≤ 0.05, **p ≤ 0.01, ***p ≤ 0.001). R: responders, NR: non-responders, HR MDS: High-risk MDS

Initially, we compared the genomic methylation profiles between the R and NR groups. To reduce noise in the data, an average methylation ratio for adjacent genomic bins was calculated using the Savitzky-Golay filter (described in materials and methods). The assignment of chromosomal bins provided a standardized approach for comparing methylation patterns both within and across patient samples. Rs exhibited a consistent increase in methylation across the genome following HMA treatment, with log_10_ methylation ratios ranging from 0.1 to 0.2. In contrast, NRs displayed either unchanged methylation levels or a decrease (log_10_ methylation ratio ≅ -0.1) post-HMA treatment (Fig. 5A).

We also compared the methylation status of each genomic bin between the two groups, implementing the Mann-Whitney U-test to evaluate the statistical rigor of our findings. In the most statistically significant bins, Rs show slight variations in methylation levels, whereas NRs exhibit substantial increases, up to 100-fold (Fig. 5B). To further deepen our analysis, chromosomal bins were searched for sequences representing genes, either protein coding or non-coding RNA species. Results are summarized in Table 2.

**Table 2:** Genes and non-coding RNAs identified within statistically significant genomic bins. Predicted and alternative gene transcripts and hairpin miRNAs are not presented in this table. Also, different isoforms of the same lncRNA are aggregated under a single representation

Genomic Bin	Protein coding Genes	Non-coding RNAs-circRNAs	Non-coding RNAs-miRNAs	Non-coding RNAs-lncRNAs
chr1_bin24	SKI, MORN1, RER1	hsa_circ_0007120, hsa_circ_0009371, hsa_circ_0009373, hsa_circ_0009376, hsa_circ_0009377, hsa_circ_0009378, hsa_circ_0009379, hsa_circ_0009372, hsa_circ_0009374, hsa_circ_0009375	Not found	lnc-SKI, lnc-PEX10
chr4_bin492	Not found	Not found	Not found	lnc-CWH43
chr8_bin858	REXO1L2P	Not found	Not found	lnc-ATP6V0D2
chr19_bin363	ZNF565, ZNF146	Not found	Not found	lnc-CAPNS1, lnc-ZNF146, lnc-COX7A1
chr19_bin364	ZFP14, ZFP82	hsa_circ_0050766, hsa_circ_0050767	Not found	lnc-ZNF146, LINC00665, lnc-ZFP14
chr21_bin83	CDC27P9, RNA28SN2, RNA18SN2, RNA5-8SN2	Not found	hsa-miR-6724-1-5p, hsa-miR-6724-2-5p, hsa-miR-10401-5p, hsa-miR-10401-3p, hsa-miR-3648	lnc-KCNE1B, lnc-SMIM11B
chr21_bin85	RNA45SN3, CDC27P10, RNA28SN1, RNA45SN1, RNA18SN1, RNA5-8SN1	Not found	hsa-miR-6724-5p, hsa-miR-10401-5p, hsa-miR-10401-3p, hsa-miR-10396b-5p, hsa-miR-10396b-3p	lnc-KCNE1B, lnc-SMIM11B
chrX_bin1159	Not found	Not found	Not found	DANT1, lnc-PLS3, DANT2, lnc-LRCH2

Most genes identified encode for ncRNA species, including circular, micro-RNAs (miRNAs) and long non-coding RNAs (lncRNAs). Among the protein-coding genes of Table 2, the Sloan-Kettering Institute proto-oncogene (*SKI*) gene located in chr1_bin24, holds a pivotal role in moderating Transforming Growth Factor-beta (TGF-β) signaling pathway. Previous studies have highlighted the important role of *SKI* in managing chronic TGF-β signaling, further affecting stem cell fitness by influencing aberrant splicing. Dysregulation of the *SKI*-TGF-β signaling axis may influence the spliceosome function and alternative splicing events, and thus provide a link to underpinning aberrant splicing patterns observed in MDS(Muench et al. 2018). These findings reinforce the hypothesis that specific genomic bins may act as potential biomarkers for predicting treatment efficacy and merit further investigation to expand our knowledge on epigenetic regulation events guided by either protein coding genes or aberrant expression of non-coding RNAs.

## Discussion

DNA methylation abnormalities play a crucial role in the pathophysiology of MDS and have clear implications in diagnosis, prognosis, and treatment of the disease. The accumulation of various somatic mutations in MDS, promote their expansion over time by granting a proliferation advantage to hematopoietic progenitor cells (Stengel et al. 2021; Risques and Kennedy 2018). In addition, the perturbed epigenetic landscape is complemented by the underlying mutational background, which either pre-exists or is induced as the neoplastic hematopoietic clones continue to expand(Hunter et al. 2021). However, in contrast to genetic mutations, epigenetic alterations are potentially reversable therefore, decoding epigenetic abnormalities spread across genome, may prove to be essential for developing targeted therapeutic strategies for MDS and improving patient outcomes by integrating accessible and accurate methodologies. So far, the dysregulated DNA methylation patterns in MDS have prompted interest in epigenetic therapies aimed to reverse aberrant DNA methylation by utilizing HMAs to achieve malignant cells clearance. However, the overall response rates are ranging between 30 and 50% and this may vary among the different MDS subtypes (Zeidan et al. 2018). Response to HMA treatment may include hematological improvement, transfusion independence, marrow blast cell reduction or clearance, depth of remission, or disease stabilization with varying duration of response, depending on specific cytogenetic abnormalities (e.g., deletion of chromosome 5q, monosomy 7 etc.)(Prebet et al. 2017) or with other, as yet uncertain prognostic factors influencing the probability and duration of response.

Response assessment is typically performed by standardized criteria, considering various parameters such as blood counts, bone marrow blast cell percentage, and transfusion requirements. Current molecular-based approaches that utilize NGS platforms for whole genome or partial mutational analysis can detect and monitor aberrations in multiple genetic loci however, they are unable to detect large structural abnormalities and copy number variants as well as fusion genes, which are common in MDS. Furthermore, the clinical significance for specific sets of mutations has not yet been established(Cook et al. 2022), probably due to the inability to adapt uniform algorithms for bioinformatics interpretation of the results and quality control protocols to standardize and harmonize the methodological steps between different NGS methods and labs.

In the current study we have implemented two different advanced methodologies with high analytical sensitivity, to assess DNA methylation levels in a HR-MDS patient cohort, pre- and post-HMA treatment to identify epigenetic markers affecting response to this type of treatment. A basic component of the study addresses the need for rapid and precise results regarding response of each HR-MDS patient to HMA treatment, to benefit medical decision-making and personalized monitoring of the disease.

NGS-methylation analysis prerequisites bisulfite treatment or methylation-sensitive restriction enzyme digest prior to sequencing reactions, thus distinguishing methylated from unmethylated cytosines, and identifying differentially methylated regions (DMRs) between tested samples. MeD-seq analysis data which were interpreted in our study enabled the identification of global changes in DNA methylation that are associated with HMA treatment response. We have identified distinct DNA methylation profiles across each human chromosome between responders (Rs) and non-responders (NRs), which were manifested by uniform rates of slight hypermethylation in Rs and hypomethylation among NRs apart from chromosomes 21, X and Y (Fig. 4A, B). Findings related to chromosomes 21, X, and Y merit further investigation, as they may be influenced by the limited sample size of identified DMRs compared to other chromosomes, as well as the underrepresentation of females in the patient cohort, both of which are considered as limitations of the present study. Chromosome 16 showed the highest Cliff’s delta (δ = 0.48) indicating the highest magnitude of difference between Rs and NRs. The increased methylation post-HMA treatment could be attributed to the local chromatin environment, the repetitive element density, or pre-existing histone modifications amplifying the methylation changes.

The methylation discrepancies within chromosomal bins revealed the existence of common chromosomal sites between Rs and NRs exerting significant methylation alterations associated with HMA treatment (Fig. 5A, B). This kind of analysis represents a powerful and comprehensive approach for studying epigenetic dysregulation in MDS patients and compares baseline and post-HMA treatment states. In particular, the identification of chromosomal bins with the highest significance between Rs and NRs encompassed several genes encoding for circular RNAs, miRNAs and lncRNAs (Table 2). These ncRNAs as part of the epigenetic regulatory compartment require further investigation to establish their potential significance as biomarkers for response to HMA treatment.

LC-MS/MS, a powerful analytical method which combines accuracy, high sensitivity and reproducibility, was implemented as an alternative method of analyzing the epigenetic alterations among the HR MDS cohort. The challenge of quantifying global levels of DNA methylation derivatives can be gauged by the low abundance of these epigenetic marks. In humans, about 1% of the total DNA bases consists of 5mdC(Clark et al. 2013) whereas, 5hmdC abundance is about 10 to 100-fold lower than that of 5mdC(Tahiliani et al. 2009; Kraus et al. 2012). 5hmdC is the first oxidation product along the active demethylation process of 5mdC to C (Fig. 2A) and has been reported as a stable epigenetic mark highly enriched within gene bodies of transcriptionally active genes, promoters and enhancers(Song et al. 2011). The global 5hmdC content is dramatically reduced in multiple human cancers(Pfeifer et al. 2014; Ficz and Gribben 2014), a sign which can potentially be associated with tumorigenesis. In the present study, DNA methylation analysis conducted by LC-MS/MS, confirmed partially corresponding results obtained from MeD-seq. NR patient group exhibited significantly reduced levels of 5mdC (*p* = 0.029) globally across the genome, whereas Rs displayed insignificant differences post-HMA treatment (Fig. 2C). Compared to healthy controls at baseline, Rs were significantly hypomethylated (*p* = 0.014) (Fig. 2B). On the other hand, MeD-seq chromosome methylation mapping in NRs revealed a total average towards hypomethylation with an imbalanced distribution across chromosomes and insignificant hypermethylation in Rs (Fig. 4B).

Among both Rs and NRs, the epigenetic signature of 5hmdC was only rarely detected in contrast to healthy controls, whose DNA comprised 5hmdC mark universally (Fig. 2E) and with no exception. The discovery of 5hmdC, the first product of the 5mdC oxidation by the α-ketoglutarate-dependent DNA dioxygenases (TET1-3) (Fig. 2A), as an epigenetic unit disrupted the simplicity of the traditional epigenetic paradigm and led to a re-evaluation of the DNA methylation landscape. Distribution of 5hmdC in the genome has shown enrichment within promoters, enhancers and transcriptionally active genes indicating a distinct biological role from 5mdC (He et al. 2021), which is typically considered as a repressing epigenetic mark. However, methods assessing the presence of DNA methylation sites globally, such as the NGS technology combined either with methylation-sensitive restriction enzymes or with bisulfite treatment, are unable to discriminate between 5mdC and 5hmdC(Huang et al. 2010). Our findings highlight the absence of 5hmdC in about 37.5–40% among HR MDS patient samples, a characteristic biomarker also observed in other malignancies (Pfeifer et al. 2014). This result suggests an impairment of the active DNA demethylation pathway catalyzed by the TET family of enzymes (Fig. 2A).

The interpretation of our data provide clear evidence for the qualitative and quantitative methylation alterations across genomic DNA in HR MDS, which can be summarized within the following observations: (a) the already significant hypomethylated DNA status among HR-MDS patients at baseline (prior treatment) (Fig. 2B), (b) the response of NRs to HMA-treatment by further lowering their DNA methylation values (Figs. 2C and 4A and B), suggesting a potential negative complication associated with the HMA treatment in NRs, (c) approximately 60% of the HR MDS patients have a dysfunctional DNA demethylation pathway via oxidation reactions catalyzed by the TET enzymes (Fig. 2E), (d) apart from DNA methylation status, epigenetic deregulation post-HMA treatment is further anticipated by the altered methylated levels of several sets of ncRNAs (Table 2) that may promote their aberrant expression.

Expanding the possibilities of LC-MS/MS analysis we have assessed and compared the [dA]/[T] deviation between the R and NR patient group and DNA samples from the healthy donors. Theoretical background refers to Chargaff’s rule, who defined the base pair equality: A% = T% and G% = C% for the double-stranded DNA molecules(Elson and Chargaff 1952). Certain repetitive sequences, regions with high mutation rates or highly methylated CpG islands may exhibit deviations from the expected 1:1 [dA]/[T] ratio. This is particularly relevant in epigenetics, since methylation of cytosine at CpG dinucleotides is an important epigenetic mark involved in gene regulation. Deamination of 5mdC can lead to changes in DNA methylation patterns and gene expression regulation simultaneously with the appearance of mutated sequence. Beyond this spontaneous process, the frequency of such mutational patterns produced in the genome under the frame of specific disorders is also important, as it contributes to genetic variation and can have implications in tissue homeostasis, including development or disease progress (Fig. 3A). Estimated [dA]/[T] ratio was 1.02 in healthy controls and ranged between 0.727 − 0.633 (< 1) among HR MDS. [dA]/[T] differences pre- and post-HMA treatment were insignificant (Fig. 3B). Conclusively, an extensively mutated genomic background was demonstrated by both Rs and NRs prior to HMA treatment, which seems unrelated to HMA mechanism of action.

To overcome the substantial epigenetic heterogeneity of MDS at clinical presentation, disease progression, and treatment response, high resolution methods for discriminating MDS patients eligible for HMA treatment and response assessment are vital. LC-MS/MS and MeD-seq methylation analysis utilized in the present study allowed for the characterization of discrete epigenetic features within the MDS patient cohort, providing novel insights with diagnostic, prognostic, or predictive value for the HMA response assessment. Additionally, some strengths and limitations arising from each methodology are also considered: (a) MeD-seq provides higher resolution and genome-wide coverage compared to LC-MS/MS, which estimates global methylation levels, (b) LC-MS/MS can distinguish between different epigenetic marks (5mdC and 5hmdC), while MeD-seq provides information for both marks as 5mdC, (c) MeD-seq generally has higher upfront costs due to NGS performance, but it offers higher throughput and greater information content per sample. Large scale MeD-seq also requires computing power and high expertise for bioinformatics analysis. The logistical and technological complexity involved in data processing and analysis of LC-MS/MS methodology, although is high, can overcome this limitation and be applied in the clinical setting as a valuable tool for quantifying global methylation levels to gain comprehensive insights into DNA methylation dynamics during HMA therapy.

## Conclusions

Response to hypomethylating treatment in HR MDS patients is not associated with global DNA hypomethylation, rather than with significant methylation reduction across specific chromosomal regions, which mainly include genes encoding for various ncRNA molecules. Moreover, additional hypomethylation emerging in NR samples of HR MDS patients apparently does not lead to their clinical phenotype improvement but probably stimulates disease progression. Our results highlight the impaired DNA demethylation pathway manifested by the absence of 5hmdC mark among most of the HR-MDS samples and the GC→AT mutation overload of both NRs and Rs. These observations highlight new epigenetic features underlying response to HMA therapy in HR MDS patients and merit further investigation. Finally, LC-MS/MS technology acquires all those advantages for first-line HR-MDS monitoring, to provide rapid, accurate and cost-effective results (compared to NGS) on the broad molecular background translatable into responsive/non-responsive phenotypes to HMA treatment, and consideration for inclusion in MRD concept.

## Data Availability

The raw MeD-seq data for 13 MDS samples pre- and post-HMA treatment are available at the SRA database under: PRJNA1075483.
